# The Green Tea Component (-)-Epigallocatechin-3-Gallate Sensitizes Primary Endothelial Cells to Arsenite-Induced Apoptosis by Decreasing c-Jun N-Terminal Kinase-Mediated Catalase Activity

**DOI:** 10.1371/journal.pone.0138590

**Published:** 2015-09-16

**Authors:** Jee-Youn Kim, Ji-Young Choi, Hyeon-Ju Lee, Catherine Jeonghae Byun, Jung-Hyun Park, Jae Hoon Park, Ho-Seong Cho, Sung-Jin Cho, Sangmee Ahn Jo, Inho Jo

**Affiliations:** 1 Department of Molecular Medicine, Ewha Womans University School of medicine, Seoul, South Korea; 2 Department of Radiation Oncology, College of Medicine, Yonsei University, Seoul, South Korea; 3 Department of Nanobiomedical Science & BK21 PLUS NBM Global Research Center for Regenerative Medicine, Dankook University, Cheonan, Chungnam, South Korea; 4 Department of Pharmacology, College of Pharmacy, Dankook University, Cheonan, Chungnam, South Korea; 5 Department of Pathology, College of Medicine, Kyung Hee University, Seoul, South Korea; 6 Biosafety Research Institute and College of Veterinary Medicine, Chonbuk National University, Jeonju, Chonbuk, South Korea; 7 Department of Biology, College of Natural Sciences, Chungbuk National University, Cheongju, South Korea; Rutgers, the State Univesity of New Jersey, UNITED STATES

## Abstract

The green tea component (-)-epigallocatechin-3-gallate (EGCG) has been shown to sensitize many different types of cancer cells to anticancer drug-induced apoptosis, although it protects against non-cancerous primary cells against toxicity from certain conditions such as exposure to arsenic (As) or ultraviolet irradiation. Here, we found that EGCG promotes As-induced toxicity of primary-cultured bovine aortic endothelial cells (BAEC) at doses in which treatment with each chemical alone had no such effect. Increased cell toxicity was accompanied by an increased condensed chromatin pattern and fragmented nuclei, cleaved poly(ADP-ribose) polymerase (PARP), activity of the pro-apoptotic enzymes caspases 3, 8 and 9, and Bax translocation into mitochondria, suggesting the involvement of an apoptotic signaling pathway. Fluorescence activated cell sorting analysis revealed that compared with EGCG or As alone, combined EGCG and As (EGCG/As) treatment significantly induced production of reactive oxygen species (ROS), which was accompanied by decreased catalase activity and increased lipid peroxidation. Pretreatment with N-acetyl-L-cysteine or catalase reversed EGCG/As-induced caspase activation and EC toxicity. EGCG/As also increased the phosphorylation of c-Jun N-terminal kinase (JNK), which was not reversed by catalase. However, pretreatment with the JNK inhibitor SP600125 reversed all of the observed effects of EGCG/As, suggesting that JNK may be the most upstream protein examined in this study. Finally, we also found that all the observed effects by EGCG/As are true for other types of EC tested. In conclusion, this is firstly to show that EGCG sensitizes non-cancerous EC to As-induced toxicity through ROS-mediated apoptosis, which was attributed at least in part to a JNK-activated decrease in catalase activity.

## Introduction

Arsenic (As), especially As trioxide, has long been used in pesticides, herbicides, and insecticides, although its applications are declining. Such agricultural applications have contributed to As release and contamination of the environment including the food supply and drinking water. Because ~140 million people worldwide are at risk of exposure to excessive levels of naturally occurring As in well water and groundwater [1], exposure of As in drinking water is a serious public health problem. Several studies have shown that chronic exposure to As is associated with increased incidence of several human diseases including cardiovascular disease [2]. Although the molecular mechanism in which As causes vascular disease has not yet been defined, it is well accepted that increased toxicity of vessels cells resulting from As-induced reactive oxygen species (ROS) is significant. Furthermore, several studies suggest that As decreases nitric oxide (NO) bioavailability in endothelial cells (EC) [3,4], suggesting another putative mechanism underlying As-induced increase in vascular disease. In addition to ROS and NO, several other signaling pathways are reportedly involved in As toxicity associated with vascular instability and subsequent development of vascular disease, namely, the coordinated regulation of decreased angiopoietin-1 secretion and increased secretion of vascular endothelial growth factor by vascular pericytes that surround EC [5], and alteration of intracellular calcium homeostasis in EC [6].

(-)-Epigallocatechin-3-gallate (EGCG), a major catechin constituent of green tea, has been reported to induce ROS-mediated damage of numerous types of cancer cells, potentiating the effect of anti-cancer drugs on cancer cell apoptosis. For example, EGCG highly induces ROS in colorectal carcinoma cells, but not in normal cells such as HEK293 and lung cells [7]. This result has led to the application of EGCG to selectively sensitize cancer cells to anti-cancer therapy. To this end, the combination of EGCG has been used to significantly enhance apoptotic cell death induced by manufactured anti-cancer drugs such as paclitaxel and cisplatin [8–10]. Recently, combined therapy of EGCG together with the chemopreventive polyphenol curcumin has been applied to further inhibit the growth of PC3 prostate cells [11] and treatment-resistant breast cancer cells [12]. In addition to anti-cancer chemotherapeutic agents, the ROS-generating toxicant As was also reported to enhance ROS generation activity and subsequent apoptosis of human myeloma cells when combined with EGCG [13]. Although EGCG induces ROS in cancer cells leading to cancer cell apoptosis, it is generally known to act as an anti-oxidant. EGCG protects normal, non-cancerous cells from numerous insults such as toxicants, hypoxia, and irradiation [14–16]. On the other hand, some studies have also found no such benefits of EGCG [17]. Although a detailed mechanism remains to be elucidated, the cell type-specific anti-oxidant and pro-oxidant properties of EGCG are reportedly attributable to its susceptibility to auto-oxidation and production of ROS, particularly H_2_O_2_ [18, 19].

We recently reported that As induces apoptosis of non-cancerous primary-cultured EC [4]. In this study, we investigated whether EGCG could protect against As-induced EC apoptosis. Rather unexpectedly, we found that the combination of EGCG with As (EGCG/As) further enhances ROS generation and apoptosis in EC, which was mediated at least in part by activation of a c-Jun N-terminal kinase (JNK)—catalase—ROS—caspase signaling axis.

## Materials and Methods

### Reagents

Minimum essential medium (MEM), newborn calf serum (NCS), penicillin-streptomycin, L-glutamine, Medium 199 (M199), Medium 200 (M200), Dulbecco’s phosphate-buffered saline (DPBS), Low Serum Growth Supplements (LSGS), antibiotic–antimycotic antibiotics and trypsin-EDTA solution were obtained from Gibco-BRL (Grand Island, NY, USA). Fetal bovine serum (FBS, HyClone) was purchased from GE Healthcare Life Sciences (South Logan, UT, USA). EGCG, As, N-acetyl-L-cysteine (NAC), bovine liver catalase, heparin, 4’, 6-diamidino-2-phenylindole dihydrochloride (DAPI) and MG132 were purchased from Sigma Chemical Co. (St. Louis, MO, USA). The specific JNK inhibitor SP600125 and pan-caspase inhibitor (Boc-D-FMK) were purchased from Calbiochem (San Diego, CA, USA). 2’, 7’-dichlorofluorescin diacetate (DCFH-DA) and specific fluorescent mitochondrial dye Mitotracker Red CMXRos (Mitotracker) were purchased from Molecular Probes (Eugene, OR, USA). Antibodies against cleaved poly(ADP-ribose) polymerase (PARP, Cat. No. #5625), JNK (Cat. No. #9252) and phosphorylated JNK at Thr^183^/Tyr^185^ (p-JNK, Cat. No. #9251) were obtained from Cell Signaling Technology (Beverly, MA, USA). Antibodies against Bax (Cat. No. ab7977), catalase (Cat. No. sc-50508) and β-actin (Cat. No. sc-1616) were purchased from Abcam (Cambridge, UK, USA) and Santa Cruz Biotechnology (Santa Cruz, CA, USA), respectively.

### Cell culture and treatments

Bovine aortic EC (BAEC) were isolated and maintained in MEM supplemented with 5% NCS, 2 mM L-glutamine, and antibiotics (penicillin, 100 U/ml, and streptomycin, 50 μg/ml) at 37°C under 5% CO_2_ in air as described previously [20]. Human umbilical vein EC (HUVEC) were isolated from fresh newborn umbilical cord vein by collagenase digestion using the standard protocol as described [21]. Before HUVEC isolation, written informed consent was obtained from each woman who donated an umbilical cord. The Institutional Review Board (IRB) of the Ewha Womans University Mokdong Hospital approved this study. HUVEC were grown on 0.2% gelatin-coated plates in M200 supplemented with LSGS, 10% FBS, and antibiotics at 37°C under 5% CO_2_. Human brain microvascular EC (HBMEC) were purchased from Cell Systems Corporation (Kirkland, WA) and grown on attachment-coated plates in CS-C complete serum free medium (Cell Systems) or in M199 supplemented with 20% FBS, 3 ng/ml recombinant human fibroblast growth factor-basic (EMD Millipore, Temecula, CA), 5 U/ml heparin, penicillin (100 U/mL), and streptomycin (100 μg/mL; Gibco-BRL) in a humidified atmosphere of 5% CO_2_ at 37°C. The cells were confirmed by their typical cobblestone configuration when viewed by light microscopy and a positive indirect immunofluorescence test for von Willebrand factor VIII. For all experiments, EC were used between passages 5 and 8, washed with PBS, and treated with EGCG, As or EGCG/As in each corresponding culture medium. In some experiments, the cells were pretreated with NAC (5 mM) 3 h before treatment. In separate experiments, catalase (50 U/ml), SP600125 (1 or 10 μM) or MG132 (20 μM) was also added 30 min prior to treatment.

### Cell viability assay

Cell viability assays were performed using 96-well plates as described previously [22]. Briefly, EC were seeded at a density of 2.5 x 10^4^ cells per well. After treatment with EGCG, As, or EGCG/As, cells were supplied with complete media containing 0.5 mg/ml of 3-(4,5 dimethylthiazol-2-yl)-2,5-diphenyltetrazoliumbromide (MTT) and incubated for 1 h at 37°C. After aspiration, 200 μl of dimethyl sulfoxide (DMSO, Sigma-Aldrich) was added to each well, and dissolved formazan product was measured at a wavelength of 570 nm.

### Caspase assay

Caspase 3, 8, or 9 activity was measured using caspase 3 and caspase 8 fluorescent assay kits (Peptron, Inc., Deajeon, Korea) and a caspase 9 colorimetric assay kit (Biosource, Camarillo, CA, USA), respectively, according to the manufacturer’s instructions. Briefly, 1 x 10^6^ cells were lysed in lysis buffer [20 mM Tris–HCl, pH7.5, 150 mM NaCl, 1% Triton X-100, 1 mM EDTA, 1 mM EGTA, 1 mM phenylmethylsulfonyl fluoride (PMSF), 10 mM β-glycerophosphate, 1 mM NaF, 1 mM Na_3_VO_4_, 1 x Protease Inhibitor Cocktail™ (Roche Molecular Biochemicals, Indianapolis, IN, USA) and centrifuged at 10,000 x g for 10 min at 4°C. The supernatant was then incubated with the appropriate caspase 3, 8 or 9 substrate. Fluorescence at 360 nm (for excitation) and 460 nm (for emission) was used to assess caspase 3 and 8 activity, while absorbance at 405 nm for caspase 9 was determined 2 h after initiation of the reaction.

### Lipid peroxidation

Lipid peroxidation was estimated by measuring the production of malondialdehyde (MDA) using the Colorimetric Microplate Assay for Lipid Peroxidation Kit (Oxford Biomedical Research, Inc., Oxford, MI, USA) according to the manufacturer’s protocol. Samples were assayed in 96-well microplates and absorbance was measured in a plate reader at 586 nm (Molecular Device, Sunnyvale, CA, USA).

### Western blot analysis

For Western blot analysis, cells treated in the absence or presence of various chemicals were washed with ice-cold Dulbecco’s PBS (DPBS) and lysed in lysis buffer. Protein concentrations were determined using the BCA protein assay kit (Sigma). Equal quantities of protein (20 μg) were separated on 10% sodium dodecyl sulfate–polyacrylamide gel under reducing conditions and then transferred onto nitrocellulose membranes. Blots were probed with the appropriate antibody directed against cleaved-PARP rabbit polycolonal (1:1000), catalase rabbit polyclonal (1:1000), JNK rabbit polyclonal (1:1000), or p-JNK rabbit polyclonal (1:1000) followed by anti-rabbit horseradish peroxidase-conjugated IgG (1:5,000; Santa Cruz, Cat. No. sc-2313) for secondary antibody and finally developed using enhanced chemiluminescence reagents (ECL, Amersham, Piscataway, NJ, USA). The level of β-actin expression was used as an internal control.

### Detection of intracellular ROS levels

Production of intracellular ROS was measured using the oxidation-sensitive fluorescent probe DCFH-DA, which is based on the ROS-dependent oxidation of DCFH-DA to 2’,7’-dichlorofluorescein (DCF) as described previously [4], with a minor modification. Briefly, cells treated with EGCG, As, or EGCG/As were trypsinized and collected by centrifugation at 1000 x g for 5 min. The cells were then washed in PBS, resuspended in 500 μl of PBS containing 5 μM DCFH-DA, and then incubated at 37°C for 30 min. Cells were washed again with PBS and monitored by FACSCalibur flow cytometry (BD Bioscience, San Jose, CA) at an excitation wavelength of 488 nm and an emission wavelength of 525 nm. ROS levels were assessed by comparing the changes in fluorescence intensity in chemical-treated cells with that of control treated cells.

### Immunocytochemistry

After culture on coverslip coated with poly-L-lysine, BAEC were treated with Mitotracker (Molecular Probes, Inc.), at a final concentration of 400 nM. After 20 min of incubation, the cells were harvested and washed three times with phosphate-buffered saline (PBS). The cells were fixed with 4% paraformaldehyde in PBS and permeabilized with 0.1% Triton X-100. After blocking with 3% bovine serum albumin in PBS, the cells were incubated with anti-Bax antibody (1:250) for 2 h at room temperature. The cells were then stained with a secondary antibody conjugated with fluorescein isothiocyanate (FITC) and subsequently stained with DAPI and viewed under confocal microscopy (META 510, Carl Zeiss, Inc., Jena, Germany).

### Assay for antioxidant enzyme levels

The activity of catalase and superoxide dismutase (SOD) was measured with a catalase spectrophotometric assay kit (Oxis International Inc., Foster City, CA, USA) and SOD assay kit-WST (Dojindo Laboratories, Kumamoto, Japan), respectively, according to the manufacturer’s instructions.

### Statistical analysis

All statistical analyses were performed using SPSS software ver. 21 (SPSS Inc., Chicago, IL, USA). Data are expressed as the mean ± standard deviation (S.D.). One-way ANOVA followed by *post hoc* Student-Newman-Keuls test was used for analysis of data; different characters were used to show significance at *P* < 0.05.

## Results

### Combined EGCG/As treatment induces apoptosis in BAEC

**Fig 1:**
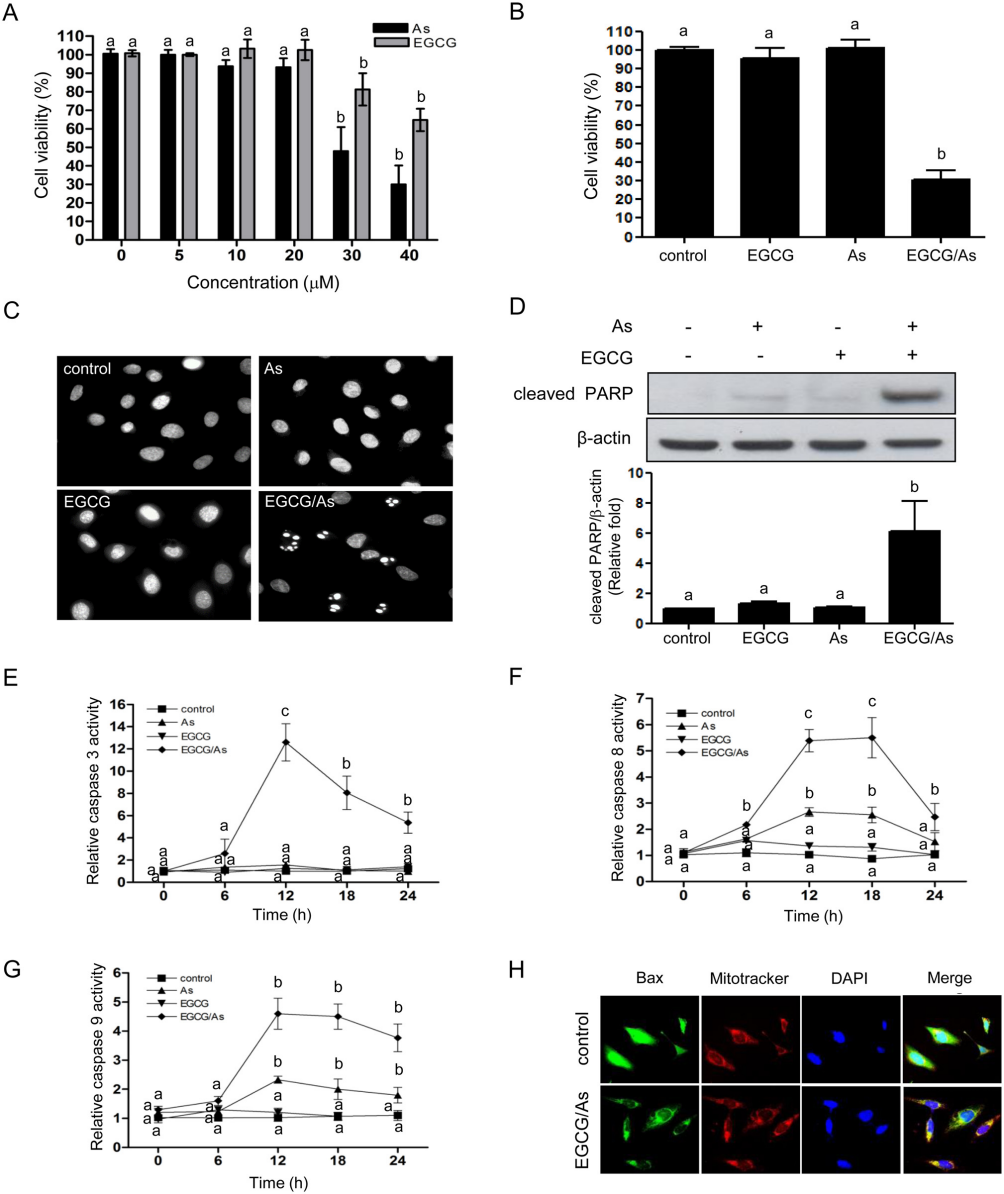
Combined EGCG/As treatment increases apoptosis in BAEC. (A) BAEC were treated with various doses (0, 5, 10, 20, 30, or 40 μM) of As or EGCG for 24 h. (B) In some experiments, cells were also treated for 24 h with 20 μM EGCG, 20 μM As, or the combination of 20 μM EGCG and As each (EGCG/As). (A, B) Cell viability was measured using MTT assay. (C, D) Cells treated with EGCG, As, or EGCG/As for 12 h. (C) Apoptotic cells were detected by DAPI staining. (D) Cells were lysed in RIPA buffer. An equal amount (20 μg) of each cell lysate was subjected to Western blot analysis. Levels of cleaved PARP expression were detected with an anti-cleaved PARP antibody. Quantifications were performed using densitometry (Image J software) and results were normalized to β-actin. (E-G) The activity of caspases (3, 8, and 9) was measured in cells treated with EGCG, As or EGCG/As for the specified times (0, 6, 12, 18, or 24 h). All line graphs represent the relative caspase activity of the control. (H) Assay for Bax translocation into the mitochondria. Cells treated with EGCG, As, or EGCG/As for 12 h were stained with FITC-conjugated anti-Bax antibody, Mitotracker as a marker of mitochondria, or DAPI. All bar graphs represent the mean ± S.D. of 3 independent experiments. The different characters refer to significant differences (*P <* 0.05) among groups, which were determined by one-way ANOVA followed by post hoc Student-Newman-Keuls analysis.

To determine whether EGCG protects against As-stimulated toxicity in BAEC, cells were first treated with various doses (0, 5, 10, 20, 30 or 40 μM) of EGCG alone for 24 h. Unexpectedly, however, no protective effect of EGCG was found under our conditions, with high doses (30 and 40 μM) decreasing EC viability. We found no effect at low doses (0, 5, 10 or 20 μM) (Fig 1A). As expected, treatment with a high dose (30 or 40 μM) of As also significantly decreased EC viability. Because of the apparently toxic effect of high doses of EGCG on viability of primary non-cancerous EC like human myeloma cells, we tested whether EGCG potentiated As-induced toxicity in EC. As shown in Fig 1B, EGCG/As significantly decreased cell viability to ~ 30% (of control; Fig 1B) at the dose (each 20 μM) of As or EGCG that did not affect cell viability. DAPI staining results analyzed by fluorescence microscopy revealed apoptotic cells with condensed chromatin patterns and fragmented nuclei in the EGCG/As-treated group but not the others (Fig 1C). Apoptosis in EGCG/As-treated BAEC was further supported by a demonstrable increase in PARP cleavage (Fig 1D), while there was little increase of cleaved PARP in either As- or EGCG-treated cells. We next tested whether EGCG/As-induced apoptosis was associated with changes in activity of pro-apoptotic enzymes such as caspases (3, 8, or 9) and BAX translocation. As shown in Fig 1E, caspase 3 activity was dramatically increased (~13 fold of control) at 12 h and then gradually decreased (~5 fold of control) at 24 h in the EGCG/As-treated cells, while either As or EGCG alone did not affect caspase 3 activity. Furthermore, treatment with EGCG/As also dramatically increased (~5 fold of control) caspase 8 activity in a time-dependent manner up to 18 h (Fig 1F). This increase dropped to ~2.4 fold of control at 24 h. Under these experimental conditions, As alone (20 μM) also increased caspase 8 activity at 12 to 18 h but to a lesser extent (~ 2 fold of control). Similarly, treatment with EGCG/As significantly increased caspase 9 activity (~ 5 fold of control) up to 24 h, although a ~2 fold increase of caspase 9 activity was detected in As-treated cells (Fig 1G). Finally, the translocation of Bax from the cytosol into mitochondria was only detected in EC in response to EGCG/As treatment (Fig 1H). Taken together, these findings suggested that although low dose (20 μM) of As may induce modest apoptosis through activation of caspases 8 and 9, this effect is dramatically potentiated when combined with EGCG.

### Combined EGCG/As treatment increases ROS production and decreases catalase activity

**Fig 2:**
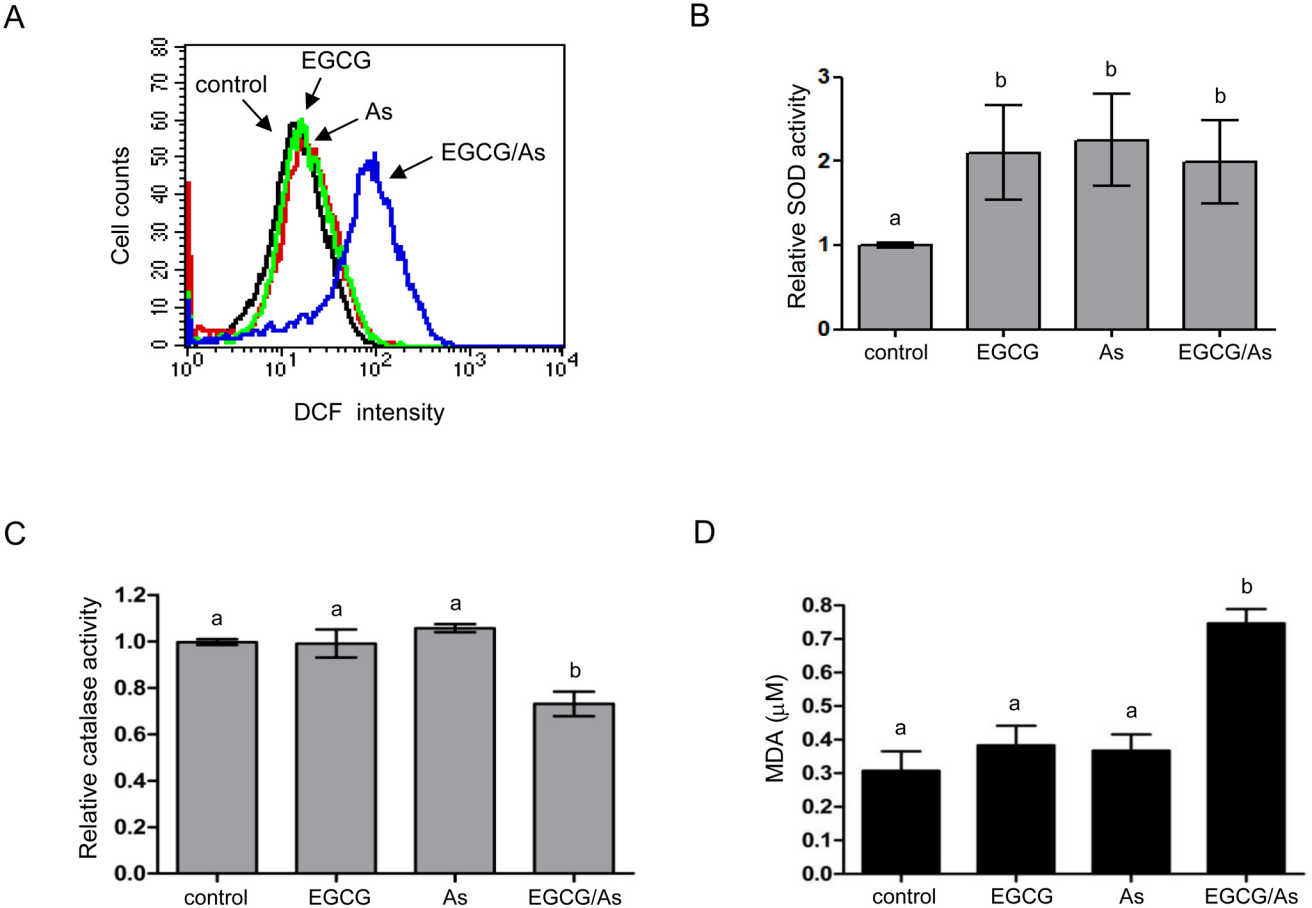
Combined EGCG/As treatment increases ROS generation and decreases the activity of catalase but not SOD. (A) ROS levels were determined by flow cytometric analysis using DCFH-DA staining. Cells were treated with EGCG, As, or EGCG/As (each 20 μM) for 3 h and then stained with DCFH-DA. Stained cells were analyzed by flow cytometry using Cellquest software. The data are representative of 3 independent experiments. (B) SOD activity was assessed in cells treated with EGCG, As, or EGCG/As (each 20 μM) for 30 min. (C) The catalase activity was measured in EC treated with EGCG, As, or EGCG/As (each 20 μM) for 2.5 h. (D) Lipid peroxidation was estimated by measuring the production of malondialdehyde (MDA) using the Colorimetric Microplate Assay for Lipid Peroxidation Kit (Oxford) according to the manufacturer’s protocol. All bar graphs represent the mean ± S.D. of 3 independent experiments. Statistical analysis was accomplished as described in the legend of Fig 1.

Previously, enhanced apoptosis by the combination of each low dose of EGCG/As was reported to be associated with the generation of ROS in malignant B cells [23]. We also investigated whether ECGC/As was associated with ROS production in non-cancerous BACE. Compared to cells treated with either As or EGCG alone, combined treatment for 3 h significantly increased ROS levels in BAEC (Fig 2A). This result promptly led us to examine whether EGCG/As also affected the activity of antioxidant enzymes in EC such as SOD or catalase, which can regulate cellular ROS production. As shown in Fig 2B, treatment with either As, EGCG, or EGCG/As increased SOD activity when compared with control, suggesting that SOD may not be involved in EGCG/As-specific increase of ROS production in EC. However, we observed a significant decrease in catalase activity in EC treated with EGCG/As (Fig 2C), suggesting a role for catalase in this signaling pathway. Because excessive ROS generates hydroxyl radical (·OH) causing peroxidation of lipids, we measured the concentration of MDA, which is known as a final production of lipid peroxidation in cells. As shown in Fig 2D, only the combined EGCG/As treatment significantly increased MDA concentration. Taken together, these results suggested that EGCG/As generates ROS, which is likely the result of decreased catalase activity.

### EGCG/As-induced apoptosis is reversed by antioxidant agent NAC and catalase

**Fig 3:**
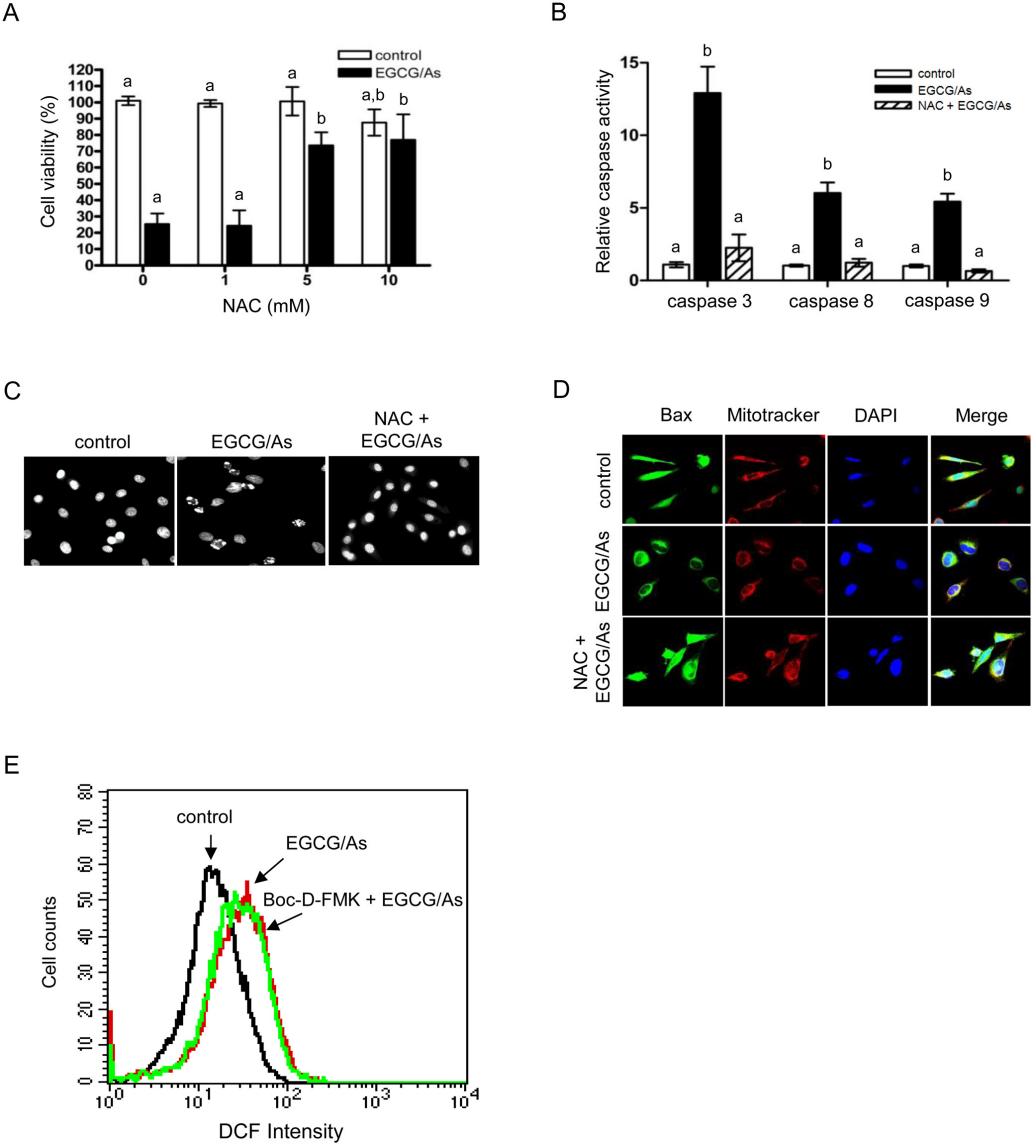
NAC reverses cytotoxicity and pro-caspase activity induced by combined EGCG/As treatment. (A) BAEC were pretreated with various doses (0, 1, 5, or 10 mM) of NAC for 3 h prior to EGCG/As treatment for 24 h. (B-D) In separate experiments, EC were pretreated with the indicated dose (5 mM) of NAC. (E) In flow cytometric analysis, BAEC were pretreated with 20 μM Boc-D-FMK for 3 h prior to EGCG/As treatment for 12 h. (A) Cell viability, (B) caspase activity, (C) DAPI staining, (D) Bax translocation into the mitochondria, and (E) flow cytometric analyses were performed as described in the legend of Figs 1 and 2. All bar graphs represent the mean ± S.D. of 3 independent experiments. Statistical analysis was accomplished as described in the legend of Fig 1.

**Fig 4:**
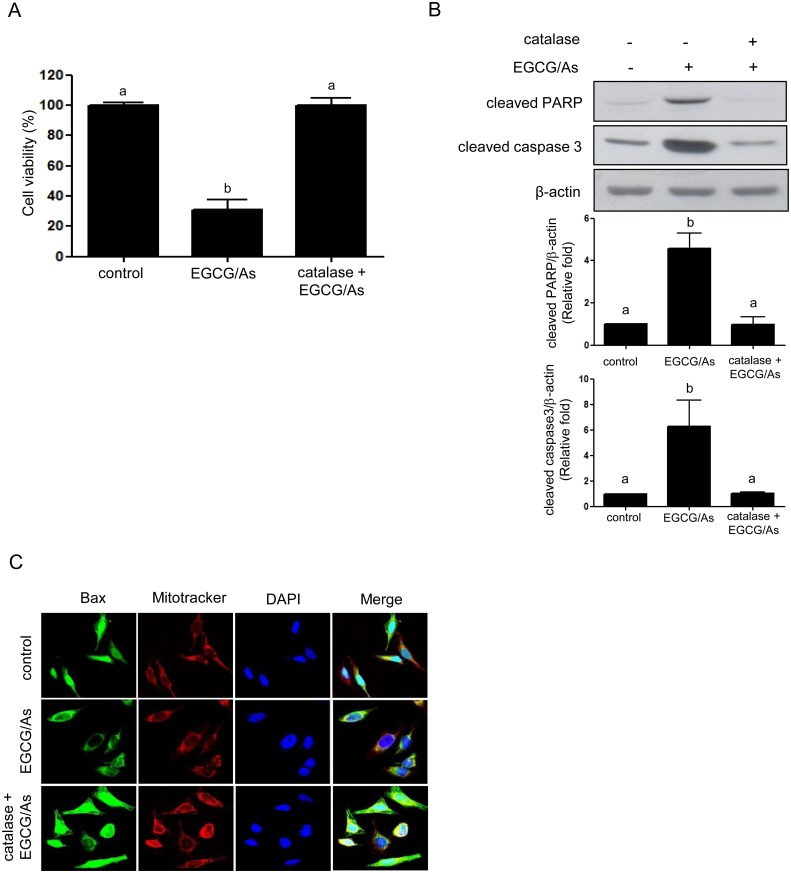
Catalase reverses cytotoxicity and pro-caspase activity induced by combined EGCG/As treatment. BAEC pretreated with catalase (50 U/ml) for 30 min were exposed to EGCG/As for 24 h. (A) Cell viability, (B) Western blot analysis using the indicated antibodies, and (C) Bax translocation into the mitochondria were determined as described in the legend of Fig 1. All bar graphs represent the mean ± S.D. of 3 independent experiments. Statistical analysis was accomplished as described in the legend of Fig 1.

Next, in an attempt to verify whether EGCG/As-derived apoptosis is indeed mediated by ROS, we examined whether the well-known antioxidant NAC rescues cells from all of the observed effects of EGCG/As treatment. As shown in Fig 3A, pretreatment with NAC (5 or 10 mM) significantly rescued (~80% of control) cell viability following EGCG/As treatment. Maximum rescue was found in cells pretreated with 5 mM NAC, and was not further improved by treatment with 10 mM NAC. Therefore, all subsequent experiments were performed using 5 mM NAC. NAC also significantly blocked the EGCG/As-stimulated activities of all of the caspases tested in this study (Fig 3B). DAPI staining experiments revealed that the condensed chromatin pattern and fragmented nuclei, as an index of apoptosis, associated with EGCG/As treatment disappeared when cells were pretreated with 5 mM NAC (Fig 3C). Furthermore, Bax translocation into mitochondria by EGCG/As was also significantly inhibited by 5 mM NAC (Fig 3D). However, we found that the pan-caspase inhibitor Boc-D-FMK did not restore the EGCG/As-stimulated increase in ROS production (Fig 3E), indicating that ROS production is upstream of caspase activation. Because EGCG/As significantly decreased the activity of catalase (Fig 2C), we next examined whether catalase was indeed involved in ROS-derived EC apoptosis as a result of EGCG/As treatment. As shown in Fig 4A, pretreatment with catalase (50 U/ml) almost completely restored the EGCG/As-induced decrease in cell viability. Furthermore, Western blot analysis also showed that catalase attenuated the cleavage of PARP and activation of caspase 3 by EGCG/As treatment (Fig 4B). Finally, EGCG/As-stimulated Bax translocation into mitochondria was also inhibited by catalase (Fig 4C). Thus, all of these results clearly indicated that catalase plays an important role in mediating EGCG/As-stimulated EC apoptosis.

### JNK mediates EGCG/As-stimulated apoptosis by decreasing protein level and activity of catalase

**Fig 5:**
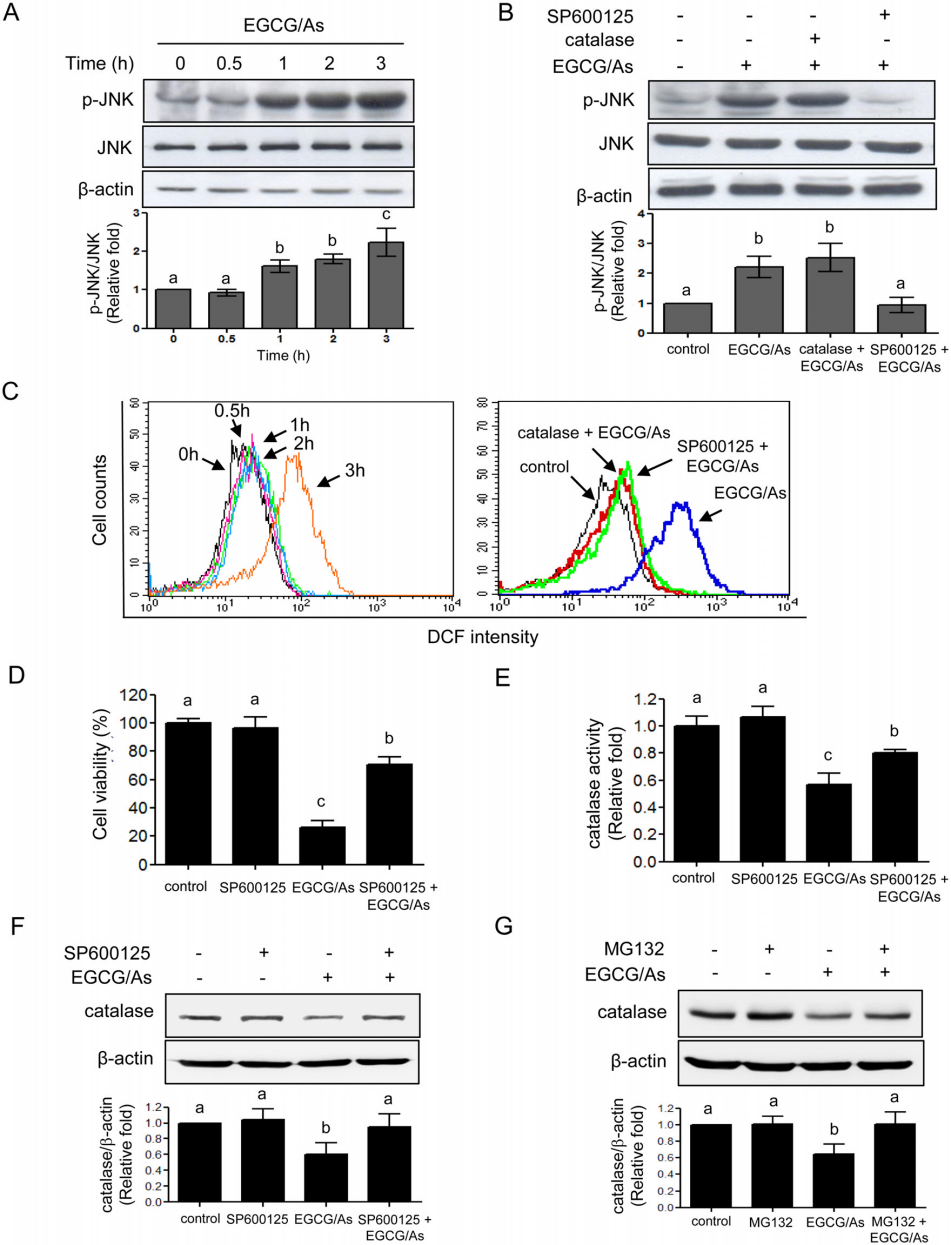
JNK mediates catalase activity, ROS production, and apoptosis altered by combined EGCG/As treatment. (A) BAEC were treated with EGCG/As for the indicated times (0, 0.5, 1, 2, or 3 h). (B) After pretreatment with catalase (50 U/ml) or the JNK inhibitor SP600125 (1 μM) for 30 min, EC were treated with EGCG/As for 1 h. The level of phosphorylated JNK (p-JNK) and total JNK protein was detected by Western blot analysis. (C) Cells were prepared and stained as described in the legends of Fig 5A and Fig 2. In some experiments, cells were pretreated as described in the legend of Fig 5B, followed by treatment with EGCG/As for 3 h. Flow cytometric analysis was performed as described in the legend of Fig 2. (D) Cell viability was determined as described in the legend of Fig 1 using BAEC pretreated with SP600125 prior to EGCG/As treatment for 24 h. (E) Catalase activity was measured as described in the legend of Fig 2 using BAEC pretreated with SP600125 prior to EGCG/As treatment for 2.5 h. (F, G) Cells were prepared, and pretreated with SP600125 (F) or MG132 (20 μM) (G) for 30 min prior to treatment of EGCG/As for 2.5 h. Cell lysate (30 μg) was subjected on 10% SDS-PAGE, and the level of catalase protein was then detected as described in **Materials and methods**. All bar graphs represent the mean±S.D. of 3 independent experiments. Statistical analysis was accomplished as described in the legend of Fig 1.

JNK is known as a major mediator that plays an important role in inducing apoptosis in a variety of cells [24, 25]. Using SP600125, a pharmacological inhibitor of JNK, we tested whether JNK also played a role in the EGCG/As-derived apoptosis. Western blot analysis revealed that EGCG/As increased phosphorylation of JNK at Thr^183^/Tyr^185^ (p-JNK) in a time-dependent manner. The significant increase of p-JNK was detected at 1 h after treatment with EGCG/As, and this effect increased gradually up to 3 h (Fig 5A). As expected, SP600125 (1 μM) completely blocked the EGCG/As-stimulated increase in p-JNK. However, scavenging of ROS by catalase did not alter the level of p-JNK induced by EGCG/As (Fig 5B), suggesting that ROS generation may not be upstream of JNK in the EGCG/As-stimulated EC apoptotic pathway. Based on this result, we next examined whether JNK was upstream of EGCG/As-mediated ROS production. As expected, pretreatment with catalase reversed ROS production induced by EGCG/As treatment (Fig 5C). Furthermore, increased ROS production by EGCG/As was almost completely reversed by pretreatment with SP600125, suggesting that JNK is indeed upstream of ROS production. Moreover, the EGCG/As-induced decrease in cell viability was also significantly reversed (~70% of control) in EC pretreated with SP600125 (Fig 5D). Interestingly, SP600125 also restored the EGCG/As-induced decrease in catalase activity, despite not altering catalase activity in basal EC (Fig 5E). The latter finding was further confirmed by showing that EGCG/As significantly decreased the level of catalase protein under our experimental conditions, which was reversed by pretreatment with SP600125 (Fig 5F). This finding suggested that JNK activation is the most upstream signaling event in the EGCG/As-induced EC apoptosis pathway; this process is mediated by decreasing protein level and activity of catalase. Lastly, we also found that pretreatment with MG132 (20 μM) (Fig 5G) reversed the decreased catalase protein level by EGCG/As, suggesting a role for proteasomal degradation.

### Combined EGCG/As treatment causes the toxicity of other types of EC, HUVEC and HBMEC, via a mechanism similar to that in BAEC

**Fig 6:**
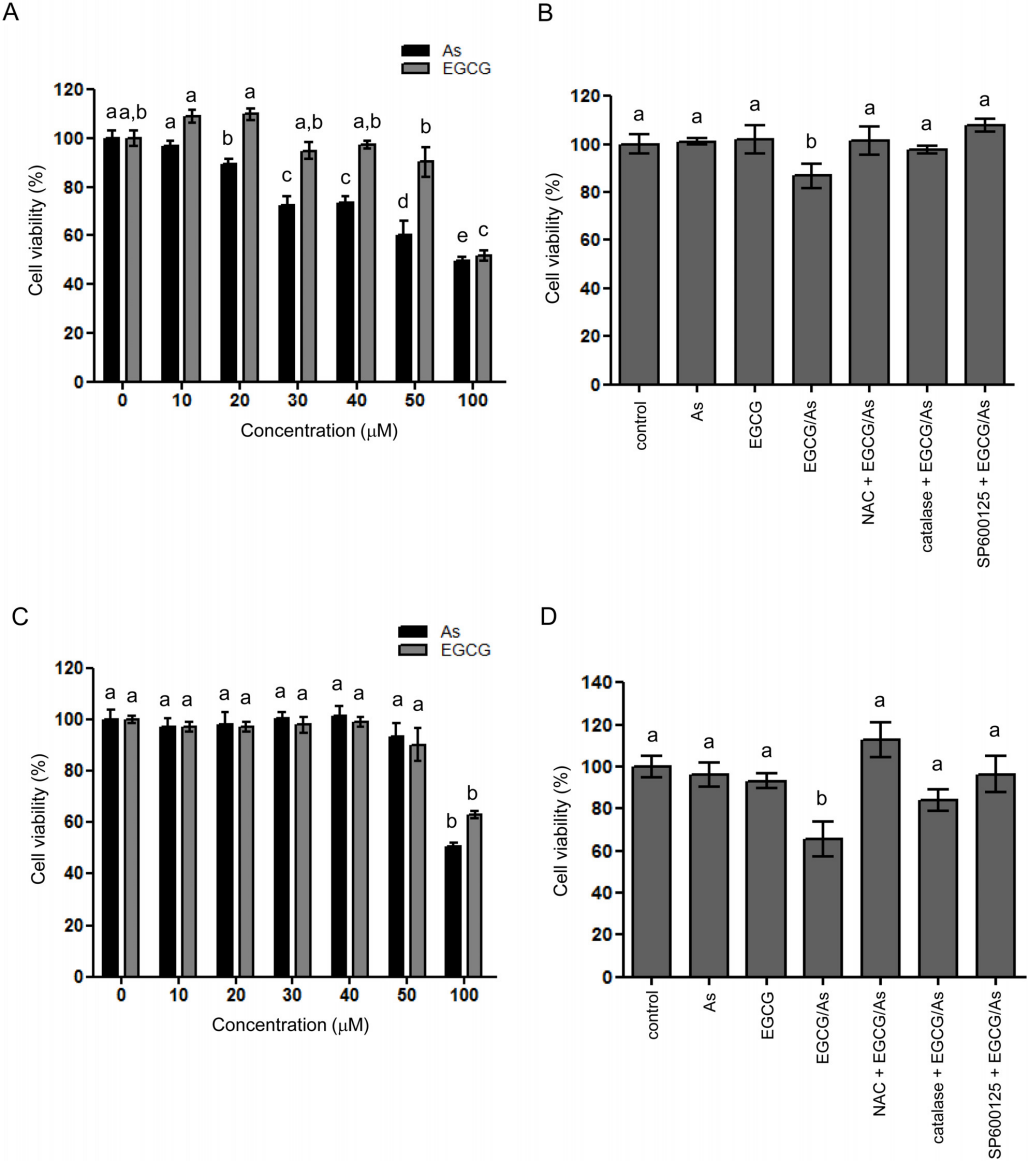
Combined EGCG/As decreases the viability of two types of EC, HUVEC and HBMEC. (A) HUVEC were prepared and treated with various doses (0, 10, 20, 30, 40, 50 or 100 μM) of As or EGCG for 24 h. (B) In separate experiments, HUVEC were also treated with 50 μM EGCG, 10 μM As, or the combination of 50 μM EGCG and 10 μM As (EGCG/As) for 24 h. In some experiments, cells were pretreated with 5 mM NAC, 50 U/ml catalase or 1 μM SP600125 for 30 min prior to exposed to EGCG/As. (C) HBMEC were prepared and treated with various doses (0, 10, 20, 30, 40, 50 or 100 μM) of As or EGCG for 24 h. (D) In separate experiments, cells were also treated with 50 M EGCG, 50 μM As, or the combination of 50 μM EGCG and As each (EGCG/As) for 24 h. In separate experiments, cells were pretreated with 5 mM NAC, 50 U/ml catalase or 10 μM SP600125 for 30 min prior to exposed to EGCG/As. Cell viability was measured using MTT assay. All bar graphs represent the mean ± S.D. of 3–5 independent experiments. Statistical analysis was accomplished as described in the legend of Fig 1.

We also tested whether the toxic effect of combined EGCG/As treatment is true for other EC. To this end, we used two primary EC, HUVEC and HBMEC. Using HUVEC, we found that As alone (for 24 h treatment) dramatically decreased HUVEC viability in a dose-dependent manner, while no significant toxicity was found in EGCG-treated cells (up to 50 μM) (Fig 6A). Only high dose (100 μM) of EGCG clearly decreased HUVEC viability (~50% of control). Based on these findings, we selected As (10 μM) and EGCG (50 μM) to examine the combined effect on EC viability. As shown in Fig 6B, EGCG/As significantly decreased HUVEC viability, which was restored by pretreatment with NAC, catalase or SP600125 (1 μM). Like HUVEC, we also found that EGCG or As alone significantly induced HBMEC toxicity, but with quite different efficacies; we found no toxic effect at low doses (up to 50 μM) on HBMEC viability, but a significant toxic effect was found at high dose (each 100 μM) (Fig 6C). Nonetheless, EGCG/As significantly decreased HBMEC viability to ~ 60% (of control; Fig 6D) at the dose (each 50 μM) of As or EGCG that did not affect cell viability. Furthermore, this decreased HBMEC viability was almost completely reversed by pretreatment with NAC, catalase or SP600125 (10 μM). Taken together, our results further supported that EGCG/As significantly induces the toxicity of other types of EC, HUVEC and HBMEC, at least in part via a mechanism similar to that in BAEC.

## Discussion

Our data demonstrate that the combination of EGCG/As treatment significantly induces EC apoptosis by increasing ROS production at a dose in which treatment of each chemical alone has no such effect. Furthermore, the increased EC apoptosis was mediated at least in part by inhibiting catalase activity via JNK activation. Although detailed clinical studies using large populations are needed before making a conclusive claim, caution may be advised against frequently drinking green tea among people routinely exposed to considerable levels of As.

Although EGCG sensitizes a variety of different types of cancer cells to apoptosis and subsequent toxicity induced by anti-cancer agents, its potentiating effect on non-cancerous primary cells remains controversial. Our study clearly showed that EGCG potentiates As-mediated toxicity of EC, non-cancerous primary cells, which is roughly consistent with several previous studies. For example, EGCG was reported to sensitize HUVEC to ischemia/reperfusion [26], rheumatoid arthritis synovial fibroblasts to tumor necrosis factor α [27], and CD4+ T cells to interferon γ [28]. Nonetheless, other studies have reported on the inhibitory effect of EGCG in As-derived cell toxicity, demonstrating that it attenuates As-induced apoptosis in mouse JB6 epidermal cells [29] and As-stimulated cytotoxicity in Chinese hamster V-79 lung fibroblastic cells [30]. Although the reasons underlying these conflicting results are not yet clear, they may be attributable to different experimental conditions including different types of cells. For example, we used EC while previous studies used epidermal cells or lung fibroblasts. In this regard, it is important to note that EGCG alone has been reported to both decrease and increase cell toxicity in a cell type- and context-dependent manner.

Because almost the same SOD activity was detected in EC treated with either As, EGCG, or EGCG/As, together with decreased catalase activity only in EGCG/As-treated EC, it is likely that the higher DCF intensity was due to a higher level of H_2_O_2_ but not of O_2_^·-^, which accumulated in EC treated with EGCG/As. Furthermore, O_2_^·-^ is generally known to be rapidly dismutated by SOD and converted to H_2_O_2_. Although H_2_O_2_ plays a role as a secondary messenger in the regulation of survival- and growth-related signaling cascades, it also results in significant cell damage when its production exceeds the capacity of the cellular anti-oxidant defense system. The role for H_2_O_2_ in inducing cell toxicity is further manifested by its capability of conversion into more toxic radicals, such as hydroxyl radicals. In most nucleated cells including osteoclastic cells, Fe^2+^, in combination with H_2_O_2_, is known to participate in Fenton reaction to generate hydroxyl radicals [13]. Although our data clearly show that EGCG/As generates H_2_O_2_, how Fe^2+^ are produced in BAEC warrants further investigation. In this regard, As was reported to contribute to Fe^2+^ production via heme degradation catalyzed by heme oxygenase -1 in HL-60 human promyelocytic cells [31].

Several previous studies have showed that JNK is a downstream signaling protein of H_2_O_2_ for inducing cell apoptosis; indeed, H_2_O_2_ induces apoptosis through the JNK pathway in SH-SY5Y human neuroblastoma cells [32], vascular EC [33], and trophoblast-like JEG3 cells [34]. Using the JNK inhibitor SP600125 and catalase, however, our present study showed clearly that JNK was upstream of H_2_O_2_ production via inhibiting catalase activity in the EC apoptotic signaling pathway (Fig 6). In support of this data, a previous study showed that SP600125 blocks the positive effect of catalase on drug transporter activity in HepG2 cells [35], indicating a potential role for JNK as an upstream protein of catalase action in this specific study. In this regard, from several previous findings, a possible mechanism underlying JNK-mediated catalase inactivation and subsequent H_2_O_2_ production was developed as follows; 1) during the apoptotic response to genotoxic stress, JNK mediates the release of cytoplasmic Abl tyrosine kinase (c-Abl) from an inactive complex with 14-3-3 [36]; 2) the released c-Abl is activated and translocates into the nucleus [37]; 3) the translocated c-Abl phosphorylates catalase at Tyr^231^ and Tyr^386^ resulting in activation of catalase; 4) simultaneously, the phosphorylation of catalase at Tyr^231^ and Tyr^386^ also promotes degradation of catalase itself via a proteasomal pathway, thereby increasing H_2_O_2_ production [38, 39]. In this study, we found that EGCG/As decreased the level of catalase protein, which was blocked by SP600125, suggesting that JNK-derived decreased catalase protein level is one possible mechanism underlying EGCG/As-mediated increase in H_2_O_2_ production in BAEC. Furthermore, our study also revealed that MG132 reversed catalase degradation by EGCG/As (Fig 5G), indicating an involvement of proteasomal pathway in the observed effects of EGCG/As. However, whether c-Abl is also involved in the effects of EGCG/As described in this study will require further experiments.

Lastly, in addition to BAEC, we found that the combined EGCG/As treatment also significantly induced the toxicity of other types of EC, HUVEC and HBMEC, at dose of As or EGCG that did not affect cell viability. However, their efficacies differed substantially. Although the reasons underlying these differences are yet to be clarified, it is speculated that these different efficacies result from the different capabilities of EC to handle ROS induced by EGCG/As. Nonetheless, pretreatment with NAC, catalase or SP600125 restores EGCG/As-induced toxicity of all three types of EC examined, providing a general mechanism.

**Fig 7:**
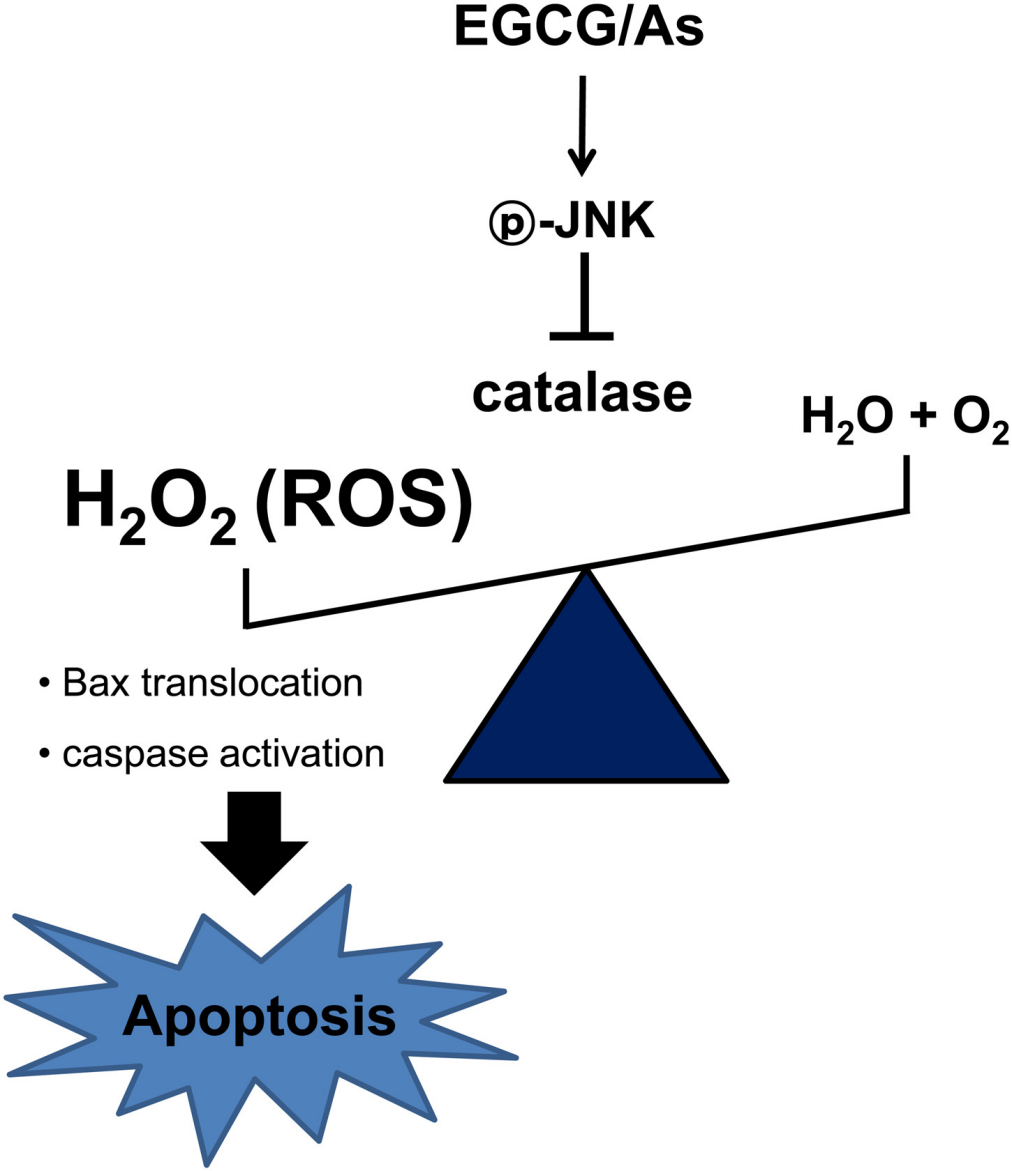
Schematic illustration of the molecular mechanism of EGCG/As-stimulated apoptosis of BAEC. (1) Combined EGCG/As treatment activates JNK. (2) Activated JNK decreases catalase activity. (3) Decreased catalase activity increases ROS (mainly H_2_O_2_) production in BAEC. (4) ROS activates pro-apoptotic machinery, consequently increasing EC apoptosis.

In conclusion, our results demonstrate for the first time that the combination of EGCG and As significantly induces EC apoptosis at doses in which treatment of each chemical alone has no such effect, and that this process is mediated by a JNK—catalase—ROS—caspase signaling axis (Fig 7). Contrary to several previous studies, we found that JNK acts as the uppermost signaling molecule in this EGCG/As-mediated EC apoptotic signaling pathway, and therefore use of a JNK inhibitor may protect from EC toxicity-related diseases such as atherosclerosis [40] and infarction [41]. Finally, although the *in vivo* relevance of our data needs to be verified using animals and human population, caution may be warranted against frequently drinking green tea among individuals exposed to As.
